# Redox-Responsive Gold Nanoparticles Coated with Hyaluronic Acid and Folic Acid for Application in Targeting Anticancer Therapy

**DOI:** 10.3390/molecules29071564

**Published:** 2024-03-31

**Authors:** Raissa Munderere, Muhammad Gulfam, Israr Ali, Seon-Hwa Kim, Trung Thang Vu, Sang-Hyug Park, Kwon Taek Lim

**Affiliations:** 1 Industry 4.0 Convergence Bionics Engineering, Pukyong National University, Busan 48513, Republic of Korea; floraissan@gmail.com (R.M.); seonhwakim431@gmail.com (S.-H.K.); 2 New-Senior Oriented Smart Health Care Education Center (BK21 Plus), Pukyong National University, Busan 48513, Republic of Korea; 3 Ashland Specialties Ireland Ltd., N91 F6PD Mullingar, Ireland; gulft1777@gmail.com; 4 Department of Smart Green Technology Engineering, Pukyong National University, Busan 48513, Republic of Korea; israrchem@gmail.com (I.A.); vutrungthang29@gmail.com (T.T.V.); 5 Major of Biomedical Engineering, Division of Smart Healthcare, College of Information Technology and Convergence, Pukyong National University, Busan 48513, Republic of Korea; 6 Institute of Display Semiconductor Technology, Pukyong National University, Busan 48513, Republic of Korea

**Keywords:** drug delivery, gold nanoparticles, dual target, methotrexate, hybrid

## Abstract

Methotrexate (MTX) has poor water solubility and low bioavailability, and cancer cells can become resistant to it, which limits its safe delivery to tumor sites and reduces its clinical efficacy. Herein, we developed novel redox-responsive hybrid nanoparticles (NPs) from hyaluronic acid (HA) and 3-mercaptopropionic acid (MPA)-coated gold NPs (gold@MPA NPs), which were further conjugated with folic acid (FA). The design of FA-HA-ss-gold NPs aimed at enhancing cellular uptake specifically in cancer cells using an active FA/HA dual targeting strategy for enhanced tumor eradication. MTX was successfully encapsulated into FA-HA-ss-gold NPs, with drug encapsulation efficiency (EE) as high as >98.7%. The physicochemical properties of the NPs were investigated in terms of size, surface charges, wavelength reflectance, and chemical bonds. MTX was released in a sustained manner in glutathione (GSH). The cellular uptake experiments showed effective uptake of FA-HA-ss-gold over HA-ss-gold NPs in the deep tumor. Moreover, the release studies provided strong evidence that FA-HA-ss-gold NPs serve as GSH-responsive carriers. In vitro, anti-tumor activity tests showed that FA-HA-ss-gold/MTX NPs exhibited significantly higher cytotoxic activity against both human cervical cancer (HeLa) cells and breast cancer (BT-20) cells compared to gold only and HA-ss-gold/MTX NPs while being safe for human embryonic kidney (HEK-293) cells. Therefore, this present study suggests that FA-HA-ss-gold NPs are promising active targeting hybrid nanocarriers that are stable, controllable, biocompatible, biodegradable, and with enhanced cancer cell targetability for the safe delivery of hydrophobic anticancer drugs.

## 1. Introduction

Methotrexate (MTX) is an extensively used potent chemotherapy drug for treating various cancers and autoimmune and inflammatory diseases such as rheumatoid arthritis, Crohn’s disease, multiple sclerosis, and psoriasis. MTX acts as an anticancer agent by inhibiting dihydrofolate reductase, preventing the conversion of dihydrofolate to tetrahydrofolate, which is required for the synthesis of DNA [1]. However, its clinical effectiveness is hindered by its poor aqueous solubility (0.01 mg/mL at 20 °C) and low permeability, leading to suboptimal bioavailability. Researchers have explored diverse drug delivery systems, including silver nanoparticles (NPs), gold NPs, liposomes, cyclodextrin complexes, emulsions, nanospheres, polymeric NPs, and polymeric micelles to mitigate the side effects of chemotherapy drugs and enhance their therapeutic potential [2]. Among these approaches, polymeric and inorganic NPs have drawn significant attention due to their ability to accommodate high drug payloads, achieve sustained drug release, enhance cellular uptake, and enable targeted drug delivery to the tumor [3]. In addition, the tumor microenvironment (TME) differs significantly from normal physiological tissue, featuring extreme hypoxia, low pH, elevated levels of acidic glutathione (GSH), and increased reactive oxygen species (ROS). Given these distinctive features, designing drug delivery systems capable of responding to the TME appears to be a promising ongoing therapeutic approach [4,5,6].

The unique shapes of gold NPs, like rod shapes, spheres, nanoshells, and nanorods with sizes varying from 1 to 100 nm, as well as surface properties, inertness, high biocompatibility, stability in biological medium, and non-cytotoxicity, represent essential advantages over other metals. Gold NPs have a strong affinity for sulfurized compounds, which is very useful for drug delivery and specific targeting of deep tissues. Furthermore, the large functional surface-to-mass ratio increases efficient drug payload, avoiding the cytotoxic effects in healthy tissues caused by overdoses. Several discrete studies previously described cytotoxicity and the impact of size on toxicity, biodistribution, retention time, efficacy, and physiological response of the nanoparticles [7]. Therefore, they have attracted a lot of attention in the field of nanobiotechnology, mostly because of their unique plasmonic properties. Due to their unparalleled properties, such as absorption and efficient transport, gold NPs can be used in a variety of anticancer therapies [8,9]. Alice et al. have shown that even though gold NPs are chemically inert and, hence, are believed to remain endlessly intact in tissues, sizes of 4–22 nm can be degraded in vitro by cells [10]. In this regard, we attempted to synthesize spherical gold NPs with a size of 21 nm. In addition, modern applications of gold NPs are tremendous beyond toxicity to chemical sensing and cellular imaging of deep tissues. However, as for the cytotoxicity of gold NPs, the available in vitro data remain inconsistent, indicating either long-term cytotoxicity to normal cells, negligible toxicity to cancer cells, and cell type-dependent cytotoxic activity [11,12,13,14].

Moreover, generally, unmodified NPs after intravenous administration are very often rapidly recognized and bound by opsonins in the blood, which facilitates their phagocytosis and removal by macrophages. In addition, appropriate surface modifications have the potential to “mask” gold NPs from the reticuloendothelial system (RES), thus ensuring stability and longer blood circulation time, improving safety and biocompatibility and playing a crucial part in new advanced cancer therapies and molecular imaging of various tissues [15,16,17]. Thiol groups have a strong affinity for binding to gold surfaces, and under alkaline conditions, they can displace citrate ions, forming gold–thiol bonds [9,18]. Surface functionalization offers more stable, efficient, and controllable NPs; hence, mercaptopropionic acid (MPA), a well-known and well-studied gold capping agent, was chosen as a thiol substitute for the citrate to make gold@MPA NPs with improved stability. These MPA ligands have a hydrophilic carboxylic acid (-COOH) group that can interact with aqueous environments and a hydrophobic alkyl tail that provides some hydrophobic character, which makes them amphiphilic [19].

**Scheme 1:**
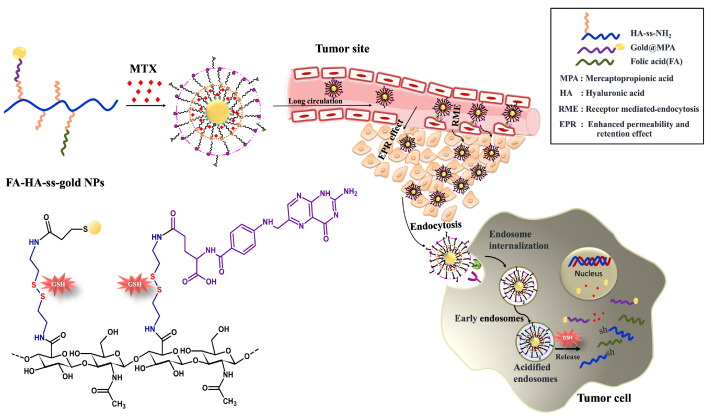
Graphical representation of dual-target and redox-responsive hybrid gold NPs.

In developing nanoparticles for cancer therapy, it is crucial to use biocompatible drug carriers that are both biodegradable and multifunctional to maximize the use of various receptors overexpressed on the surface of many cancerous cells compared to healthy cells and minimize side effects for effective therapeutics by understanding tumor microenvironment conditions [20,21,22]. In this regard, a biodegradable ligand, hyaluronic acid (HA), was selected as a polysaccharide main chain for modification due to its particular affinity for tumor cells (Scheme 1). HA as a major component of the extracellular matrix (ECM) has gained prominence in biotechnology and biomedicine due to its versatility [23,24]. It boasts several advantages for pharmaceutical applications, including its wide availability, biocompatibility, long-term blood circulation, biodegradability, non-toxicity, and non-immunogenicity, and was hence attached to coated gold NPs in this study. Additionally, HA presents abundant functional groups (-COOH, -OH) that can be modified or functionalized. HA and its derivatives are valuable ligands for targeted drug delivery because of their strong affinity with cell-specific surface markers like the glycoprotein cluster of differentiation (CD44) and the receptor for HA-mediated motility (RHAMM), which are overexpressed in many tumor types. Notably, HA plays a critical role in activating kinase pathways and controlling tumor angiogenesis [25,26]. However, using HA affinity to CD44 as the sole targeted therapy has been debated and is known to be inefficient.

**Figure 1:**
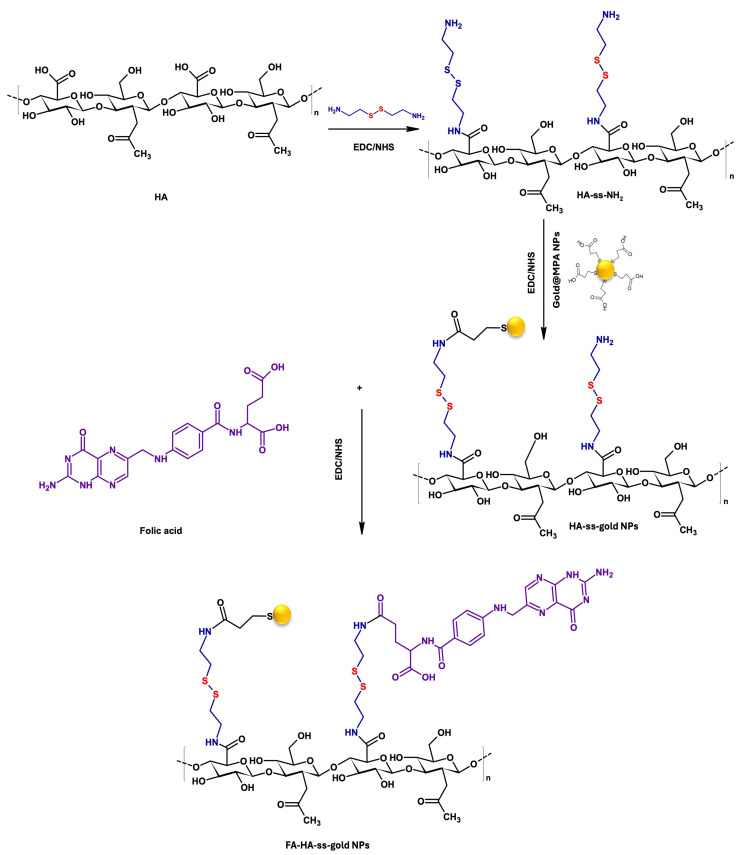
Graphical illustration of the synthesis of the redox-responsive and dual-targeting hybrid nanoparticles.

Achieving successful targeted cancer therapy remains a significant challenge. Therefore, numerous researchers have employed dual-targeted therapies as ideal treatments. This dual receptor targeting mechanism enables the precise and effective delivery of NPs to tumor cells, maximizing their therapeutic outcome while being blind to normal cells [27]. In this regard, to further enhance the targeted drug delivery efficacy of HA, we added folic acid (FA), known as vitamin B9, which is renowned for its biocompatibility, ensuring safety for normal cells while acting as a critical agent in folate receptor (FR)-targeted cancer treatments [28,29,30]. Historically, FRs were among the first to be recognized as legitimate targets for cancer therapy due to their high abundance on the surface of various cancer cells and low expression in healthy tissues [31]. Utilizing this cancer cell uptake specificity, we designed a redox-responsive hybrid system with a disulfide reducible bond (HA-ss-gold NPs), further modified with FA to achieve FA-HA-ss-gold NPs, as shown in Figure 1.

Over recent years, there have been advancements in drug delivery systems that employ disulfide linkages, targeting the induced intracellular release of drugs triggered by GSH, acting as a reducing agent to rapidly break the disulfide bonds through a thiol-disulfide conversion process. Compared to regular blood and extracellular fluid, tumor cells have intracellular GSH concentrations that are 100–1000 times higher, reaching up to 20 mM compared with normal cells (2–20 μM), which gives them distinct redox characteristics [21,32]. This unique intracellular redox potential has paved the way for the design of flexible, internal stimuli-responsive polymeric carriers. Such carriers are designed to enhance intracellular drug delivery and release, hence amplifying therapeutic efficacy [33,34]. To benefit from this strategy, cystamine with a cleavable organic disulfide bond was used as a cross-linking agent to link HA and gold due to its inherent stability, biocompatibility, and the ability to form a cage for therapeutic cargos, and it can undergo controlled cleavage in response to the elevated GSH levels of tumor environments [35].

**Scheme 2:**
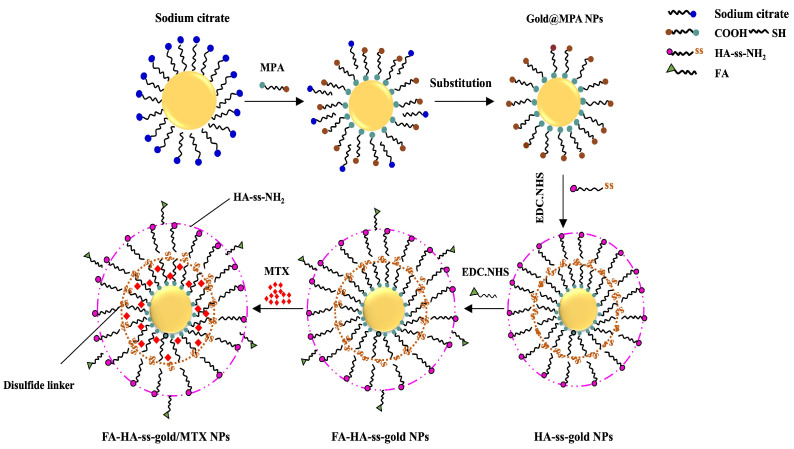
Ligand substitution and coupling chemistry involved in the synthesis of MTX-loaded hybrid gold nanoparticles (FA-HA-ss-gold NPs/MTX NPs).

The further conjugation of folic acid to the HA-ss-gold NPs could achieve dual active targeting. After that, MTX was successfully encapsulated by FA-HA-ss-gold NP through hydrophobic–hydrophobic interactions (Scheme 2). The EE and the drug-loading capacities (LC) of MTX-loaded FA-HA-ss-gold NPs were evaluated, and the MTX release from the NPs in a reduction environment was investigated. A WST-1 assay was performed to evaluate the anti-tumor efficacy of MTX-loaded NPs on HeLa and BT-20 cells. The cellular uptake was studied in HeLa cells and determined using fluorescence microscopy.

Hybrid nanoparticles have served many purposes in the advancement of modern technologies. However, the reported properties and characteristics of organic–gold hybridized systems are still insufficient. This is because gold hybrid NP properties and behavior are directly influenced by synthetic methods, so even a small alteration during synthesis can make a difference and, hence, make them difficult to control [11]. The primary aim of this study was to develop an optimized nanoparticle system that is controllable and prioritizes safety for normal cells while being cytotoxic to cancer cells. Emphasizing a high drug-loading capacity and precision tumor targeting, we aimed for efficient binding and internalization in tumor cells. The developed hybrid NPs were demonstrated to be smart nanocarriers for safe and effective cancer therapies. Detailed analyses, including physicochemical characterization, in vitro cytotoxicity assessments, and cellular uptake studies, further elucidate the potential of the developed hybrid NPs.

## 2. Results and Discussions

### 2.1. Characterization of Gold@MPA NPs

**Figure 2:**
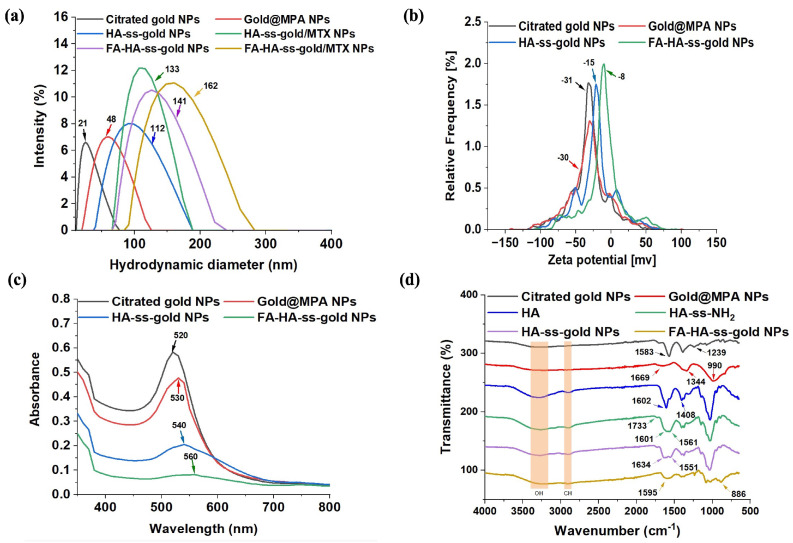
Characterization of the hybrid nanoparticles. (**a**) Hydrodynamic diameters, (**b**) zeta potential, (**c**) UV–vis spectra, and (**d**) FTIR spectra.

Aqueous solutions of gold NPs were prepared by reducing tetrachloroauric (III) with trisodium citrate. The gold NPs had an average diameter of 21 nm (Figure 2a) The formation and stability of the gold NPs were investigated using zeta potential (Figure 2b), UV–vis absorption spectroscopy (Figure 2c), and FTIR spectrum (Figure 2d). The citrated gold NPs in their original solution retained remarkable stability for more than 100 d when stored at 4 °C. However, upon purification and transfer to DI water, there was a noticeable instability in the surface of gold NPs. Therefore MPA was directly added to the synthetized gold NPs as a substitution for the citrate because it was proven to have a high affinity to the gold surface and could introduce more carboxylic groups for further functionalization [14]. Nevertheless, initial attempts at attaching MPA onto citrated gold NPs under various conditions proved unsuccessful because of the aggregation of nanoparticles. However, the efficient displacement of citrate by MPA was found to be achieved by adjusting the solution to pH 10–11 [36]. For our research objectives, we specifically targeted pre-surface functionalized gold NPs (21 nm) whose size was below 22 nm since they have been previously shown to be biodegradable at such dimensions [10]. The zeta (ξ, ZP) potential, defined as a physical–chemistry property that investigates the stability of a colloidal system, is the electrostatic potential that exists at the shear plane of a particle, which is related to both surface charge and the local environment of the particle. The ZP of the synthesized citrated gold NPs was −31 mV, with a plasmonic wavelength at 520 nm, which supported a transverse resonance mode associated with spherical NPs. The structure of gold@MPA was confirmed by a red shift observed from 520 to 530 nm in the UV–vis spectrum, indicating the presence of MPA on the gold’s surface.

In addition, the X-ray diffraction pattern (XRD) of citrated gold nanoparticles is shown in Figure S1. Several Bragg reflections with 2 θ values of 38 u, 44.43 u, 64.55 u, and 77.7 u correspond to the (111), (200), (220), and (311) planes, respectively. A significant diffraction peak (111) indicates that the network structure has grown prominently along (111) planes compared to (200). Nonetheless, the significant diffraction of the (220) peak indicates the anisotropic nature of the nanoparticles.

The FT-IR spectrum of citrated gold NPs shows characteristic bands of citric acid, where the 3274 cm^−1^ peak is assigned to OH, and 1705, 1583, and 1392 cm^−1^ peaks belong to COO stretching, while 1239 and 1082 cm^−1^ peaks belong to C-O stretching (Figure S2a). Furthermore, The FT-IR analysis of gold@MPA showed characteristic peaks at 3246 cm^−1^ assigned to O-H stretching of the alkyl groups of the alkanethiol and 1344 cm^−1^ to the C-H deformation of the alkyl group. The peak at 1669 cm^−1^ was assigned to the CO stretching of the carboxylic group of MPA, and the C–S bond peak is found at 704 cm^−1^ (Figure S2b).

### 2.2. Characterization of HA-ss-Gold NPs

**Figure 3:**
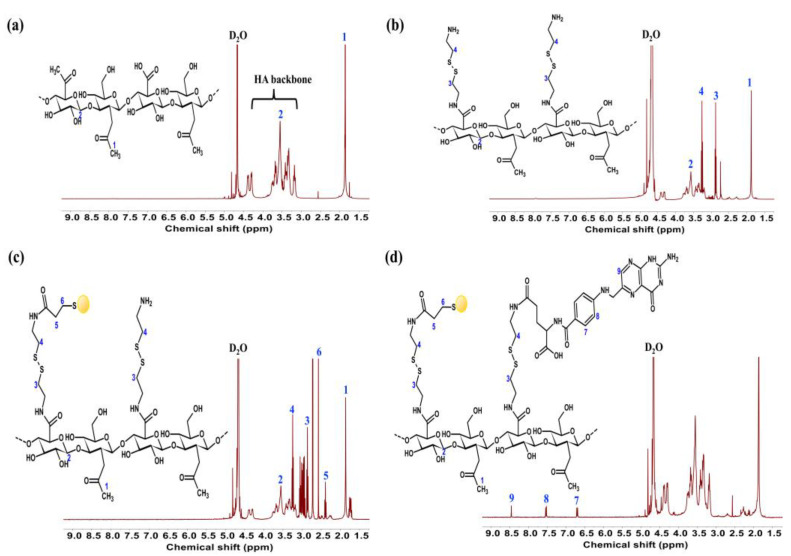
^1^HNMR spectrum characterization of (**a**) HA in D_2_O, (**b**) HA-cystamine (HA-ss-NH_2_), (**c**) HA-ss-gold NPs, and (**d**) FA-HA-ss-gold NPs.

Conventionally, HA has been altered to include thiol groups, methacrylate groups, alkyne/azide functions, or aldehyde groups to enhance its use in cancer therapy [37]. To synthesize HA-ss-gold, cystamine-modified HA (HA-ss-NH_2_) was first obtained by coupling HA carboxyl groups with the amino group of cystamine through a carbodiimide–amidation reaction (Figure 1). The DS of cystamine was calculated through the integration of the proton of the cystamine and methyl group of HA in the 1H NMR spectrum and was found to be 68% (Figure 3a,b). Then, the HA–cystamine linker was conjugated to the carboxylic group of gold@MPA to obtain HA-ss-gold NPs through an amine coupling reaction using EDC and NHS. The structure of HA-ss-gold was confirmed by FTIR (Figure S2c,d), showing the characteristic peaks at 3280 cm^−1^ for O-H stretching vibrations, 2899 cm^−1^, and 1375 cm^−1^ for C-H stretching vibrations (Figure S2e). The characteristic ^1^H NMR peaks of the N-acetyl group and hydroxyl groups in HA were found at 1.9 ppm, while the distinct peaks of methylene groups of cystamine also appeared at 3 and 3.4 ppm. Newly formed peaks of MPA were seen at 2.5 and 2.7 ppm, confirming the successful conjugation of gold@MPA NPs to HA-ss-NH_2_ (Figure 3c).

### 2.3. Characterization of FA-HA-ss-Gold NPs

For FA-HA-ss-gold NP synthesis, FA was first activated by EDC/NHS and then conjugated into HA-ss-gold NPs through an amidation reaction. The synthetic scheme is shown in Figure 1 and Scheme 2. Changes in hydrodynamic diameter throughout synthesis indicate nanoparticle structural dynamics. After coating gold NPs with MPA, the initial increase in size from 21 nm to 48 nm suggests successful surface functionalization. As a result of the subsequent attachment of HA, a substantial size increase to 112 nm occurs, primarily due to the long-chain nature of HA. As a result of encapsulating MTX in HA-ss-gold NPs, the hydrodynamic diameter increased to 133 nm. After FA conjugation to HA-ss-gold NPs, the size increased further to 141 nm and 162 nm after MTX encapsulation into FA-HA-ss-gold NPs (Figure 2a). From the zeta potential measurements (Figure 2b), the significant shift of the NPs from −31 mV to −15 mV after modification with HA confirmed successful polymer conjugation. The same negative zeta potential at all synthesis stages indicated the overall stability of the NPs in preventing particle aggregation in physiological environments, ensuring easy re-dispersion [38,39]. The reason for FA-HA-ss-gold NPs becoming less negatively charged (−8 mV) was that the more negatively charged carboxyl acid moieties (COO^−^) of HA present on the surface of the HA-ss-gold NPs were replaced by FA [40,41]. The optical properties of the nanoparticles were characterized by UV–vis spectroscopy, demonstrating that the gold NPs exhibited an ultraviolet absorption peak at 520 nm before and absorption at 530 nm after modification with MPA (Figure 2c). The absorption band was shifted to 560 nm in the final product FA-HA-ss-gold NPs.

**Figure 4:**
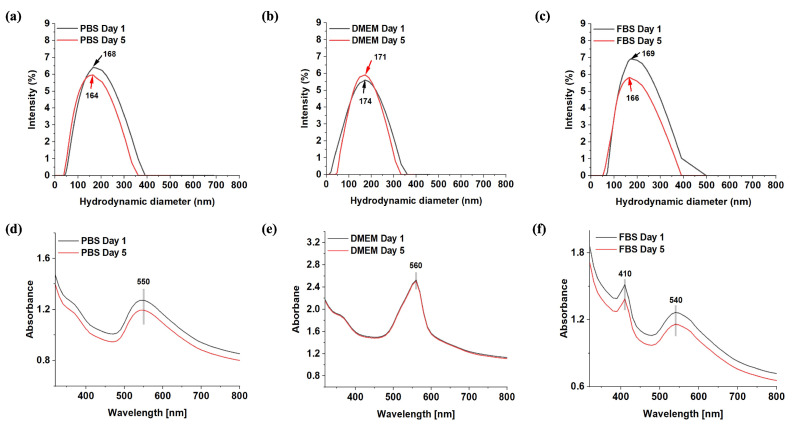
Stability investigation of FA-HA-ss-gold/MTX NPs in PBS, DMEM, and FBS by DLS: (**a**) Day 1 = 168 nm, Day 5 = 164 nm; (**b**) Day 1 = 174 nm, Day 5 = 171 nm; (**c**) Day 1 = 169 nm, Day 5 = 166 nm and by UV–vis spectroscopy: (**d**) 550 nm at Day 1 and Day 5; (**e**) 560 nm at Day 1 and Day 5; and (**f**) two peaks of 410 nm and 540 nm at both Day 1 and Day 5.

The C=C skeletal vibration at 1595 cm^−1^ is a notable FTIR peak for FA (Figure 2d). Bands at 3254 cm^−1^ represent O–H bonds of FA and HA. Amide moieties are 1409, 1378, and 1236 cm^−1^ of the Petrin ring of FA (Figure S2f). There were obvious characteristic peaks of FA at 886 cm^−1^ (Ar–H deformation vibration of the aromatic ring). Additionally, the successful grafting of FA to HA-ss-gold was further confirmed by the appearance of weak signals at the 6.6 ppm–8.6 ppm range, which corresponded to the aromatic protons of FA (Figure 3d). For the stability investigation (Figure 4), FA-HA-ss-gold NPs displayed negligible size changes and no significant changes in UV–vis spectra for Day 1 and Day 5 when dissolved, and incubated in phosphate-buffered saline (PBS), Dulbecco’s Modified Eagle Medium (DMEM), and fetal bovine serum (FBS), indicating satisfactory stability of the NPs.

Together, these complementary analyses collectively corroborate the successful synthesis of FA-HA-ss-gold NPs, providing valuable insights into the molecular structure, chemical composition, and surface chemistry of NPs.

### 2.4. Drug Loading and Release Studies of MTX-Loaded Nanoparticles

**Figure 5:**
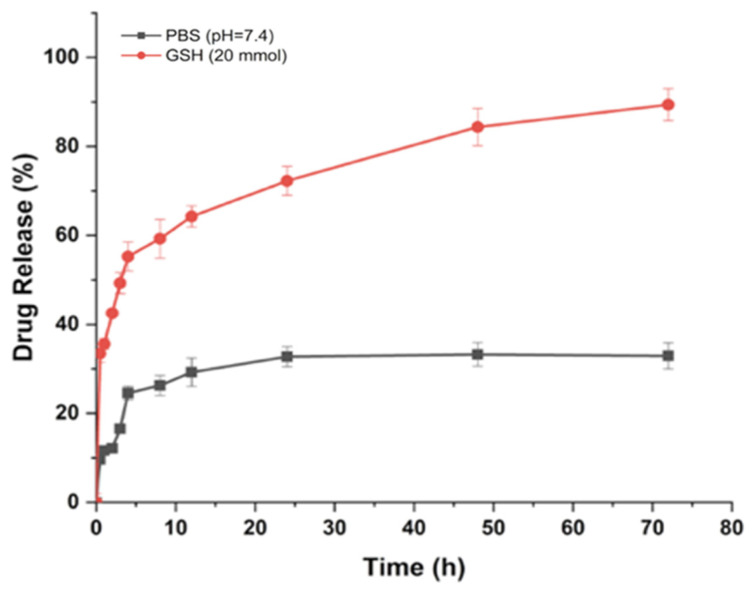
The cumulative % of MTX release profiles from dual-targeted FA-HA-ss-gold/MTX NPs in simulated physiological buffer and tumor environments.

One important metric that indicates the rationality of nano-formulations and their functionalities is encapsulation efficacy [42]. To investigate the effect of the cross-linker formulation on the drug release behavior of the nanoparticles, MTX (a joint anticancer agent) was used for drug loading and release experiments (Figure 5). The EE (%), and LC (%) of MTX-loaded FA-HA-ss-gold were found to be 98.7% and 5.5%, respectively.

The MTX release from the NPs was analyzed in PBS (pH 7.4) and 20 mmol GSH solutions as simulated physiological and tumor environments, respectively. The MTX release profiles in the PBS solution from MTX-loaded NPs are shown in Figure 5. The MTX-loaded NPs exhibit a rapid initial release of MTX in the PBS (pH 7.4) solution, which is about 31% after 24 h. In the following 72 h, they displayed a suppressed release in the physiological medium. In contrast, the release of MTX kept increasing in the reducing environment. This result can be explained by the fact that reactive GSH attacks the disulfide bond to generate thiols. This reduction reaction leads to a breakdown of the disulfide bond, allowing for the release of MTX from FA-HA-ss-gold NPs. After 72 h, approximately 86% of encapsulated MTX were released in a sustained manner.

### 2.5. In Vitro Biocompatibility

**Figure 6:**
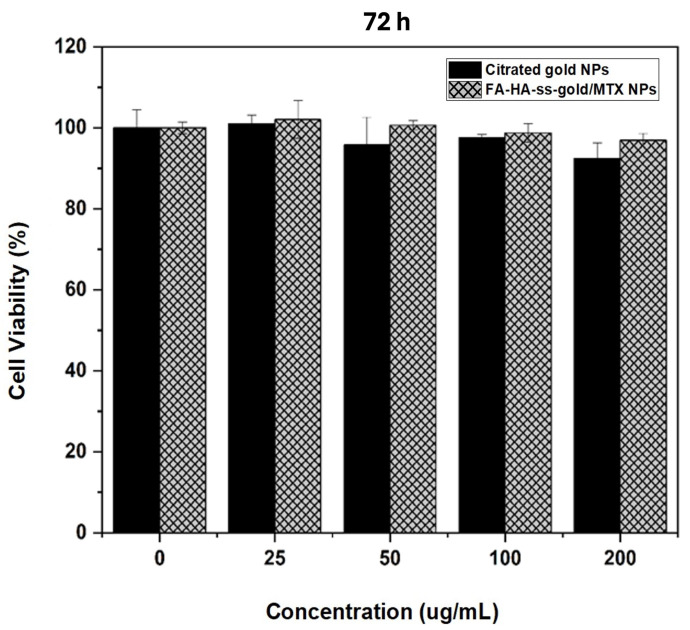
Viability assessment of Citrated gold NPs, and FA-HA-ss-gold NPS on HEK-293 cells after 72 h of treatment.

The cytocompatibility of the nanoparticles is a prerequisite for biomedical applications. The in vitro biocompatibility of FA-HA-ss-gold and gold NPs was investigated in non-cancerous cells (HEK-293 cells) using water-soluble tetrazolium WST-1 assays (Figure 6). To assess the safety of the materials, HEK-293 cells were incubated with various doses of citrated gold and FA-HA-ss-gold NPs for 48 h. The citrated gold NPs were safe at 200 µg/mL (95% cell viability). Similarly, FA-HA-ss-gold did not induce any significant cytotoxic effect (>97% cell viability) on HEK-293 cells, which is a good indication of its biocompatibility within the bloodstream and can hence mitigate side effects that are often experienced with the intravenous injection of nanoparticles during cancer therapies.

### 2.6. In Vitro Cytotoxicity

**Figure 7:**
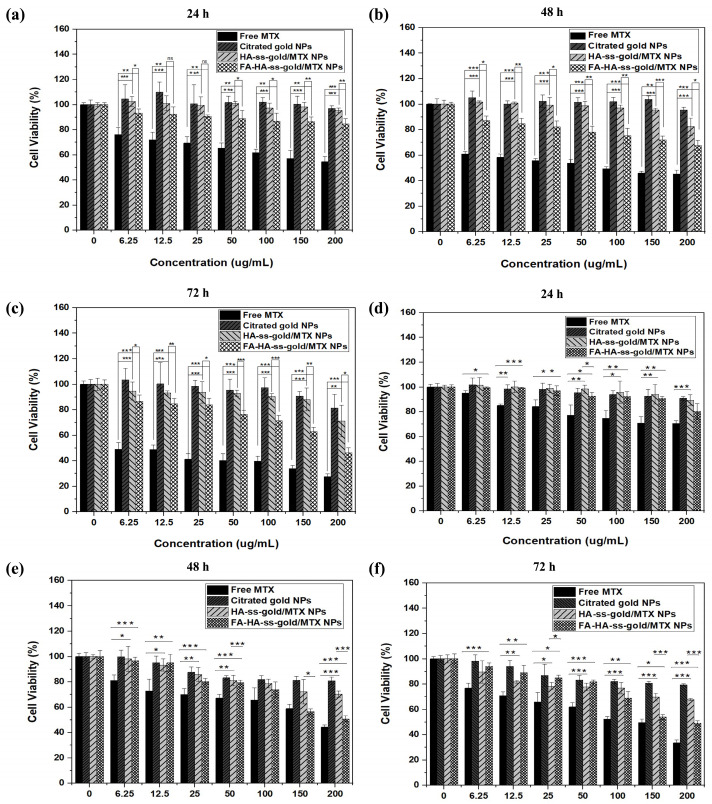
Anti-cancer assessment on HeLa cells at (**a**) 24 h, (**b**) 48 h, (**c**) 72 h, and BT-20 cells at (**d**) 24 h, (**e**) 48 h, and (**f**) 72 h treated with free MTX, citrated gold, HA-ss-gold/MTX, and FA-HA-ss-gold/MTX NPs. (*p*-value, * *p* < 0.05, ** *p* < 0.001, and *** *p* ≤ 0.0001).

To assess the cytotoxicity of FA-HA-ss-gold and HA-ss-gold NPs, WST-1 assays were performed on HeLa and BT-20 cells, and the results were compared with the free MTX group. In Figure 7, at doses greater than 100 µg/mL, FA-HA-ss-gold/MTX NPs showed a noticeable decrease of both HeLa and BT-20 cells during 24 h. Simultaneously, cells that were exposed to free MTX displayed decreased viability at every tested concentration. Citrated gold NPs alone exerted negligible cytotoxic effects, whereas HA-ss-gold/MTX NPs induced detectable cytotoxicity inferior to that of FA-HA-ss-gold/MTX NPs. The cytotoxicity effects of FA-HA-ss-gold/MTX NPs were more pronounced at the 48 h and 72 h marks, particularly at higher doses in contrast to citrated gold NPs and HA-ss-gold/MTX NPs. Moreover, a significant inhibition in the proliferation of HeLa cells at 72 h was observed upon FA-HA-ss-gold NP treatment with an IC50 value of 126.7 μg/mL, and 55.5 μg/mL for free MTX treatment. We also observed that FA-HA-ss-gold NPs caused a significant decrease in the proliferation of BT-20 cells (IC50 = 180.8 μg/mL) while free MTX was 126.7 μg/mL.

Even while free MTX groups showed a dose-dependent decrease, it is critical to draw attention to MTX’s known negative effect on normal cells, such as HEK-293, in contrast to the safer profile of FA-HA-ss-gold/MTX NPs. Furthermore, the slow release of MTX from the FA-HA-ss-gold hybrid system for extended durations ensures that MTX continuously exerts its chemotherapeutic effects.

### 2.7. Cellular Uptake Analysis

Generally, the intracellular behavior of carriers is largely influenced by their entry pathways. One prominent pathway is endocytosis, a passive targeting mechanism fundamental to eukaryote cells. Cellular uptake is an important biological response that determines the therapeutic effectiveness of any NP-based drug delivery system. This process enables cells to intake external materials by enveloping them with the plasma membrane, subsequently forming vesicles. Rhodamine-123(Rh-123) is a model compound that has been extensively studied and exhibits representative properties for positively charged active pharmaceutical ingredients that need protection against anionic barriers on the way to the in-target through the gastrointestinal (GI) tract. It has also been reported to cross membranes easily due to its lipophilic nature and accumulate in areas with negative membrane potentials, such as within the matrix of mitochondria [43]. Thus, it serves as an effective probe for nanoparticles when quantifying intracellular uptake. In this regard, to assess the cell uptake hierarchy of FA conjugated-HA-ss-gold NPs compared to HA-ss-gold NPs, Rh-123 dye was loaded into nanoparticles using the same procedure as MTX encapsulation.

**Figure 8:**
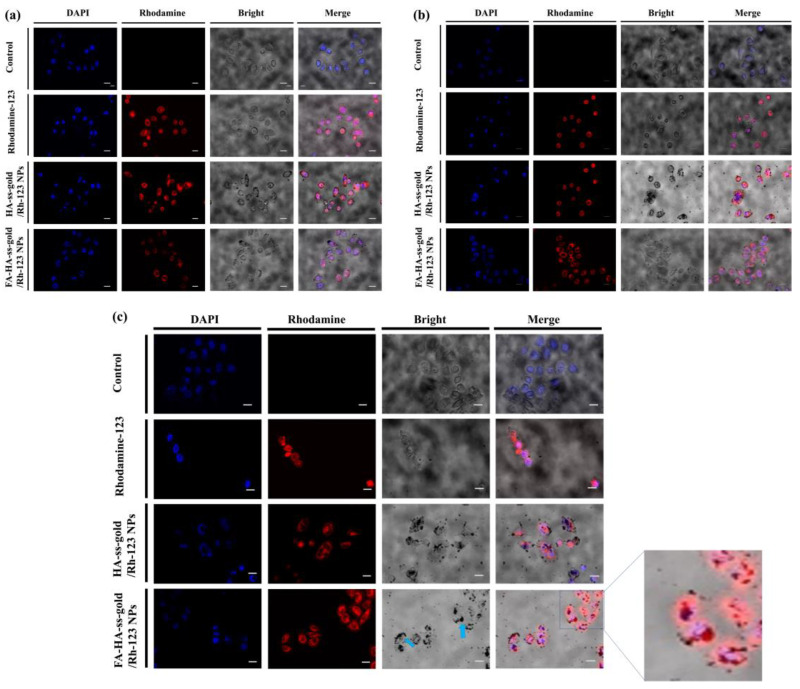
Cellular uptake and intracellular localization analysis of FA-HA-ss-gold NPs at (**a**) 3 h, (**b**) 6 h, and (**c**) 24 h with DAPI (blue), Rhodamine (Red), and the bright field. FA-HA-ss-gold NPs show the highest Rh-123 fluorescence at 24 h compared to HA-ss-gold NPs. The blue arrows show the localization of gold NPs (black dots) in the tumor. Efficiently internalized gold NPs are more observable, and most of them are found located inside the nucleus, demonstrating and proving the hierarchy of the dual-target effects of the added folic acid to HA-ss-gold NPs. This high internalization positions the FA-HA-ss-gold/MTX NPs at the frontline of biodegradable hybrid nanoparticles with multifunctional therapeutic capabilities. The scale bar is 20 μm.

Cells were stained with blue stain (DAPI dye) and initially examined in an untreated state to serve as a control. To highlight the inherent properties of Rh-123 as a fluorescent dye and show its behavior independent of nanoparticle encapsulation, cells were exposed to Rh-123 alone and analyzed. Cellular uptake analysis indicates the red fluorescence in the cells when exposed to just Rh-123, establishing the baseline behavior of the dye in these cellular environments. Furthermore, the cells were tracked using the fluorescence of Rh-123 post-treatment at 3, 6, and 24 h on HeLa cells to examine the uptake of the NPs and provide additional insight into the release behaviors. Figure 8 illustrates the fluorescence intensity of untreated cells, Rh-123 alone, and cells treated with Rh-123-loaded HA-ss-gold and Rh-123-loaded FA-HA-ss-gold NPs. The observation indicated the efficient internalization of all tested materials into the nucleus of the HeLa cells. There was an enhanced variation of the Rh-123 fluorescence intensity in the cells treated with FA-HA-ss-gold as opposed to those treated with HA-ss-gold NPs, especially evident at 24 h. These results confirm the role of FA in enhancing the safety of the nanoparticles to normal cells in terms of increased solubility, prolonged circulation time in the blood, and enhanced cellular uptake efficacy directly to cancer cells.

In addition, the bright field gives insight into the fate and localization of the NPs. There is a clear difference between Rh-123-loaded FA-HA-ss-gold and HA-ss-gold NPs compared to the control and Rh-123-only groups. At 3 h and 6 h, the dark dots seen belong to gold NPs, and this supports the previous findings that gold NPs have specific localization properties in deep tissues [14]. For the FA-HA-ss-gold group, much more gold NPs were seen inside the cell than on the cell surface after 24 h, confirming time-dependent internalization and accumulation of the NPs inside the cell, which was previously reported [44,45]. This hierarchy internalization of gold NPs by the FA group at 24 h, more so than at 3 h and 6 h and with HA-ss-gold NPs, provides an understanding of the circulation and sustained release behavior of the FA-HA-ss-gold NPs.

## 3. Experimental

### 3.1. Materials

Sodium hyaluronate (MW = 10 kDa) was purchased from Lifecore Biomedical (Chaska, MN, USA). Cystamine hydrochloride (99%), gold (III) chloride trihydrate (HAuCl_4_⋅3H_2_O), aqueous trisodium citrate dihydrate (Na_3_C_6_H_5_C_7_), MPA, FA, MTX, 1-ethyl-3-(3-dimethyl aminopropyl)-carbodiimide hydrochloride (EDC), N-hydroxysuccinimide (NHS, 98%), sodium bicarbonate (NaHCO_3_), sodium carbonate (Na_2_CO_3_), dimethyl sulfoxide (DMSO), and GSH (98%) were obtained from Sigma-Aldrich, Inc. (St. Louis, MO, USA). All the chemicals were of analytical grade and were used without purification.

### 3.2. Characterization

^1^H NMR spectra were investigated using a Fourier transform nuclear magnetic resonance spectrometer (FT-NMR 400 MHz) (JEOL/JNM ECZ-400). The chemical structure and functional groups were analyzed with a Fourier transform infrared spectrometer (JASCO/FT-4100). The spectrum was measured in transmission mode ranging from 650 to 4000 cm^−1^ wave numbers. The average particle size and zeta potential (ζ) of NPs were measured with a dynamic light scattering instrument, Litesizer500 (Anton Paar, Graz, Austria), and an X-ray diffractometer, Ultima IV (Rigaku, Tokyo, Japan) was used to measure X-ray diffraction patterns. A UV–vis spectrophotometer range was used to record UV–vis spectra with a ray diffractometer range of 250–1000 nm. The cells were grown in DMEM (HEK-293) and RPMI 1640 media (HeLa cells, and BT-20 cells), supplemented with 1% antibiotic (penicillin/streptomycin, *v*/*v*) and 10% fetal bovine serum, and incubated at 37 °C in an environment that was humidified with 5% CO_2_. Every sample was sonicated before each synthesis and characterization using an ultra sonicator (20 s ×3, 20% amplitude).

### 3.3. Gold NPs Surface Functionalization

#### 3.3.1. Preparation of Gold NPs

Gold NPs were prepared via the modified Turkevich–Frens method with slight modification [46]. In a 150 mL round bottom flask, 50 mL of aqueous HAuCl_4_·3H_2_O (0.5 mM) was heated to 100 °C. While the mixture was vigorously stirred, 2.5 mL of aqueous trisodium citrate (40 mM) preheated to 70 °C was added to the boiling precursor solution under vigorous stirring. The mixture was then stirred under reflux for 1 h, cooled to room temperature (RT), and stirred overnight at RT, resulting in gold particles with a net negative charge from the citrate ions, stabilizing the particles.

#### 3.3.2. Preparation of Gold@MPA NPs

**Figure 9:**
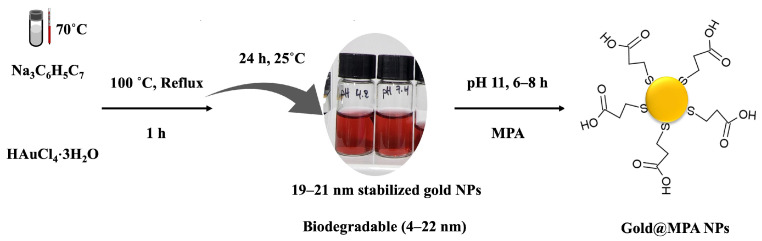
The preparation and surface modification of gold NPs.

An amount of 0.01 M of MPA was prepared in anhydrous ethanol, as shown in Figure 9. An amount of 3 mL of MPA solution was carefully added to 120 mL of gold NP solution at pH 11. The resulting solution was centrifuged, washed with 80% alcohol, and sonicated three times. The final mixture was immediately changed to pH 7, frozen for 24 h in a refrigerator, and then lyophilized for 4–5 d. The obtained product was stored at 4 °C.

### 3.4. Preparation of HA-ss-Gold NPs

HA (1000 mg, 0.1 mmol) was dissolved in DI water (25 mL). EDC.HCL (2.5 mmol) and NHS (2.5 mmol) were added, and then the mixture was stirred for 1 h to activate the carboxylic groups of HA. Then, cystamine (2.5 mmol) was neutralized to pH = 8–9 with Na_2_CO_3_, added into the mixture at 0 °C, and stirred for 24 h at RT. The formed HA-ss-NH_2_ (DS 68%) was then dialyzed against DI water for 48 h (MWCO 3500) and lyophilized. 

HA-ss-gold NPs (DS 22%) were prepared by chemical grafting of gold@MPA to HA-ss-NH_2_. Gold@MPA (22.13 mg, 0.07 mmol) was dissolved in 2.5 mL of DI water. Equimolar amounts (0.015 mmol) of EDC.HCL and NHS were added and stirred for 1h. The mixture was then dropwise added to HA-ss-NH_2_ (5 mg/mL, 0.0017 mmol) in DI water and stirred for 24 h at RT. The resulting solution was dialyzed against DI water for 48 h (MWCO 3500 Da) and frozen for 24 h in a refrigerator. Finally, the product was freeze-dried for 4 d and stored at 4 °C.

### 3.5. Preparation of FA-HA-ss-Gold NPs

To synthesize FA-HA-ss-gold NPs (DS 5%), FA (5 mg/mL) solubilized in anhydrous DMSO was reacted with an equimolar amount (0.073 mmol) of EDC.HCL and NHS. This mixture was maintained at 21 °C in a light-protected environment overnight. Subsequently, this solution was dropwise added to the HA-ss-gold (5 mg/mL, 0.008 mmol) mixture and stirred at RT for 24 h in the dark. Afterward, the resultant solution was initially dialyzed against an excess amount of NaHCO_3_–Na_2_CO_3_ buffer (pH 10) until no FA could be detected in the dialysate by measuring UV absorbance at 281 nm, and later further dialyzed against DI water for 3 d. Finally, the dialyzed solution was lyophilized and stored at 4 °C.

### 3.6. Drug Loading Analysis

The drug loading process was conducted according to the following procedure. Firstly, FA-HA-ss-gold NPs (18 mg) were dissolved in DI water to yield a 1 mg/mL concentration, sonicated, and stirred for 5 min. Subsequently, 1 mg/mL of MTX in DMSO was dropwise introduced into the NP solution using a syringe (G = 22) with gentle stirring, followed by 3 days of dialysis to remove DMSO and the unloaded MTX. The resulting solution was analyzed with UV–vis spectrophotometry (303 nm wavelength) to determine the drug loading efficiency. The percent of the drug EE and LC were calculated according to the following equations, respectively:

$$\mathrm{EE}\;\left(\%\right)=\frac{\mathrm{Amount}\;\mathrm{of}\;\mathrm{MTX}\;\mathrm{in}\;\mathrm{feed}}{\mathrm{Amount}\;\mathrm{of}\;\mathrm{MTX}\;\mathrm{used}}\times 100$$

$$\mathrm{LC}\;\left(\%\right)=\frac{\mathrm{Amount}\;\mathrm{of}\;\mathrm{MTX}\;\mathrm{in}\;\mathrm{feed}}{\mathrm{Amount}\;\mathrm{of}\;\mathrm{nanoparticles}}\times 100$$

### 3.7. Drug Release Analysis

To investigate the drug release profile, MTX-loaded FA-HA-ss-gold NPs were put in a 3500 MWCO cut-off dialysis membrane, immersed in 35 mL of either PBS (pH~7.4) or 20 mmol GSH solution, and gently agitated (100 RPM) at 37 °C. Aliquots of 3 mL were collected from vials periodically at a predetermined time interval. Additionally, a fresh solution of 3 mL of PBS or GSH was added back into each vial to compensate for the withdrawn volume. The quantity of released MTX was assessed by using a UV–visible spectrophotometer (303 nm) based on a calibration curve of MTX. The dilution factor was considered to calculate the cumulative release of DOX.

### 3.8. Cell Viability and Cytotoxicity

In 48-well plates, HEK-293 cells were cultured in a cell culture medium containing 10% FBS and DMEM at a cell density of 5 × 10^4^ cells/well. The cells were incubated for 24 h to fully attach to the well plate surface. To assess the biocompatibility of the carrier, the HEK-293 cells were treated for 72 h with different concentrations of citrated gold and FA-HA-ss-gold NPs dissolved in the cell culture medium. After 72 h, the cell culture medium was aspirated, and cells were rinsed with PBS and then treated with the WST-1 substrate (300 μL) for 30 min. After that, optical densities were measured using a microplate reader at a wavelength of 450 nm. To investigate the cytotoxicity of the prepared NPs, HeLa cells and BT-20 cells were cultured in RPMI 1640 at a cell density of 5 × 10^4^ cells/well. The cells were treated at concentrations (0, 6.25, 12.5, 25, 50, 100, 150, 200 μg/mL) of free MTX, citrated gold, HA-ss-gold/MTX, and FA-HA-ss-gold NPs for 24 h, 48 h, and 72 h. After each time point, the WST-1 assays were performed to measure the cytotoxicity of the NPs in the same way as explained above.

### 3.9. Cellular Uptake Studies

The fluorescence-based cellular uptake was performed to elucidate the effect of HA-ss-gold and FA-HA-ss-gold NPs against HeLa cells. The HeLa cells were cultured on confocal dishes and incubated for 24 h. The cells were treated with Rh-123 only, Rh-123-loaded HA-ss-gold, and Rh-123-loaded FA-HA-ss-gold NPs (immersed in cell culture medium), and then incubated for 3 h, 6 h, and 24 h at 37 °C. Subsequently, the cells were stained with Hoechst dye and analyzed using fluorescence microscopy to observe and study the cellular uptake of the nanoparticles.

## 4. Conclusions

In this paper, we prepared a novel redox-responsive hybrid nanoparticle system (FA-HA-ss-gold NPs) containing inorganic gold NPs and organic HA, further modified with FA for effective cancer drug delivery. First, the gold NPs were coated with MPA to increase stability and provide more functional groups for chemical conjugation. GSH-reducible disulfide bonds were introduced in the HA polymer and hybridized with gold-coated NPs to make HA-ss-gold NPs. To enhance the tumor-targeting ability and internalization specificity of the NPs, FA was attached to the hybrid system. MTX was successfully encapsulated in FA-HA-ss-gold NPs at >98.7% of EE (%). The designed FA-HA-ss-gold NPs had a dynamic particle size of 141.7 nm with a zeta potential of −7.8 mV and a UV absorption peak at 560 nm. The FA-conjugated NPs were found to be more internalized in the tumor cells than HA-ss-gold NPs after 24 h, giving insight into the capability of this dual-targeting system. The proximity of the localization of gold NPs in deep tumors observed in the FA-HA-ss-gold NP cellular uptake profiles could make the developed hybrid system a precise, targeted cancer therapy. The MTX-encapsulated FA-HA-ss-gold NPs ensured a sustained drug release in GSH media that mimicked the cancer environment. The toxicity of the NPs was studied in non-cancerous cells (HEK-293 cell line) and found to be safe with >90 cell viability. The overall cytotoxicity of FA-HA-ss-gold/MTX was higher than HA-ss-gold/MTX NPs against tested HeLa and BT-20 cancer cell lines, which correlates with the cellular uptake results.

Our research provides new insights into adequately designing a cost-effective hybridized gold nanoparticle system that is stable, biocompatible, and biodegradable with tunable and controllable surface chemistry for enhanced cancer therapy. These properties make this hybrid system a smart approach to developing precise cancer drug carriers for biological application not only in oncology but also in other disease models that are known to be difficult to navigate.

## Data Availability

The data presented in this study are available upon request from the corresponding author.

## Supplementary Materials

The following supporting information can be downloaded at: https://www.mdpi.com/article/10.3390/molecules29071564/s1, Figure S1. X-ray diffraction patterns of FA-HA-ss-gold NPs, Figure S2. FTIR spectra of (a) Citrated gold NPs, (b) gold@MPA NPs, (c) HA-ss-NH_2_, (d) HA-ss-gold NPs, and (e) FA-HA-ss-gold NPs.
