# Supplementary material for: Redox-Responsive Gold Nanoparticles Coated with Hyaluronic Acid and Folic Acid for Application in Targeting Anticancer Therapy
Source: Molecules. 2024 Mar 31;29(7):1564. doi: 10.3390/molecules29071564 (PMC11013442; doi:10.3390/molecules29071564)
Supplement: Supplementary file 1 [file molecules-29-01564-s001.zip › molecules-2925481-supplementary.pdf]

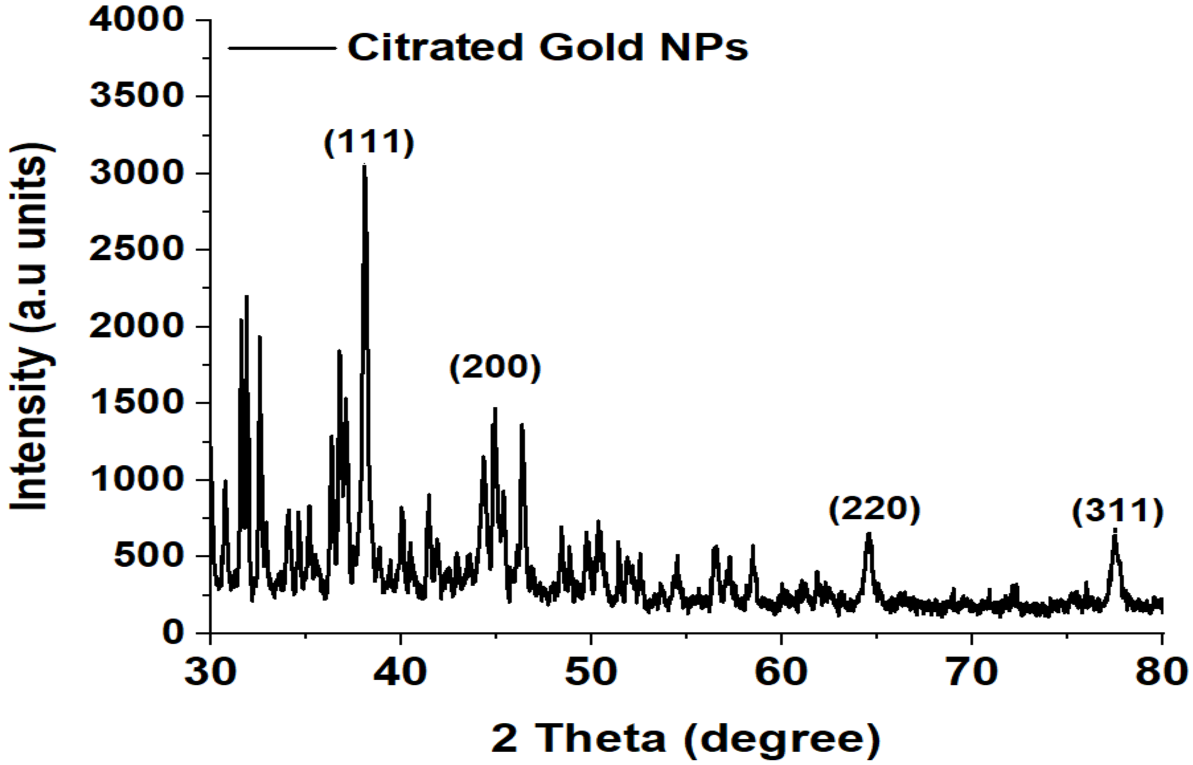

Figure S1. X-ray diffraction patterns of FA-HA-ss-Gold NPs

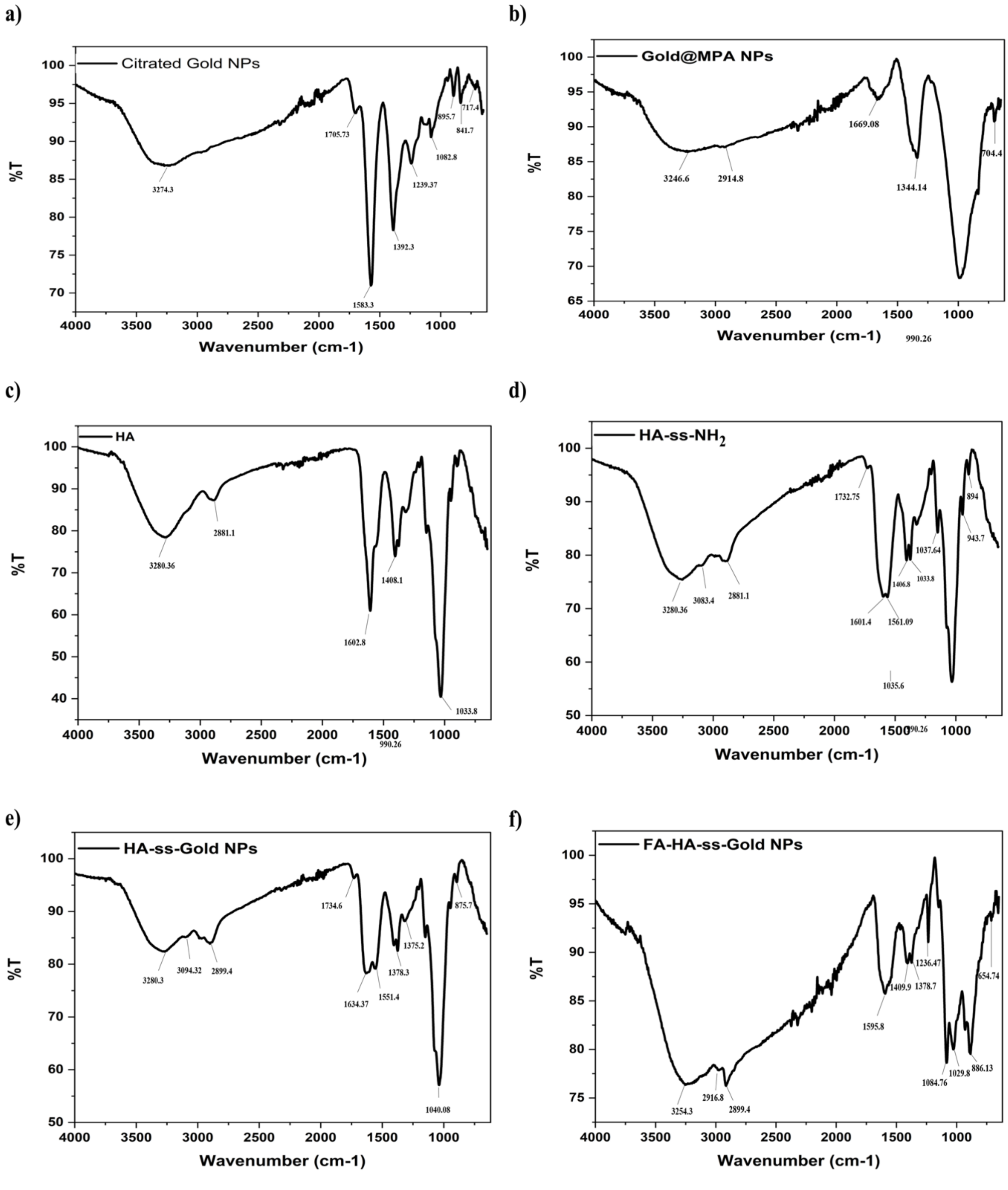

Figure S2. FTIR spectra of (a) Citrated Gold NPs, (b) Gold@MPA NPs, (c) HA-ss-NH2, (d) HA-ss-Gold NPs, and (e) FA-HA-ss-Gold NPs.
